# A New Method of Distribution of Measurement Points on Curvilinear Surfaces of Products

**DOI:** 10.3390/s19122667

**Published:** 2019-06-13

**Authors:** Marek Magdziak

**Affiliations:** The Faculty of Mechanical Engineering and Aeronautics, Rzeszów University of Technology, al. Powstańców Warszawy 12, 35-959 Rzeszów, Poland; marekm@prz.edu.pl; Tel.: +48-17-865-1491

**Keywords:** coordinate measuring technique, free-form surface, measurement strategy, contact measurements, scanning

## Abstract

The article presents the method of selecting scanning lines along which coordinate measurements, performed by using, e.g., a coordinate measuring machine working in the single point probing mode, of free-form surfaces of measured workpieces may be conducted. Additionally, the proposed method supports the user of a coordinate measuring system during defining the number of measurement points distributed along selected scanning lines, thus deciding on the final distribution of measurement points on a measured surface of a product. The proposed method enables distributing measurement points in the parts of a measured product characterized by the worst quality of manufacturing. Moreover, the new method is very automated, therefore it affects the increase in the efficiency of coordinate measurements. The effect of using the new method is the non-uniform distribution of measurement points located on free-form surfaces. The presented algorithm takes into account the lengths of selected cross-sections of a measured curvilinear surface of an object, its geometrical complexity and the accuracy of the probe radius correction process. The decision regarding the number of measurement points is made on the basis of the accuracy analysis of the calculations of the corrected measurement points obtained during the probe radius correction process and the accuracy of the substitute model representing a measured curvilinear surface. Two methods of the correction process were used. The accuracy of the applied methods of the probe radius compensation process was estimated on the basis of the deviations calculated between corrected measurement points and scanning lines. The selection of scanning lines and the number of measurement points was realized by using the expert system based on the fuzzy logic. The paper presents the results of both simulation and experimental investigations. The numerical calculations were performed for two selected free-form surfaces. The verification of the developed algorithm was carried out during experimental investigations based on a measurement of a selected free-form surface. The conducted research was aimed at verifying the correctness of the distribution of measurement points generated using the proposed method. In the case of real measurements, measurement points should be located in the places of surfaces of products characterized by the largest deviations of manufacturing. The results of the conducted investigations confirm the usefulness of the developed algorithm for defining the distribution of measurement points on curvilinear surfaces in the coordinate measuring technique. Moreover, the way of implementation of the developed method of the distribution of measurement points in selected commercial measurement software is presented, thus enabling the use of the new method in the industry.

## 1. Introduction

The coordinate measuring technique is widely used in the industrial practice in the case of measurements of products with regular geometrical shapes and workpieces built of curvilinear surfaces. Coordinate measurements can be made by using many different contact and non-contact systems [1,2]. Coordinate measuring machines (CMMs), which may be equipped with contact scanning measuring probes and probes working in the single point probing mode, are still very popular contact systems [3]. The example of a five-axis inspection machine, which is based on a CMM, enabling coordinate measurements of free-form surfaces of products is shown in the paper [4], which also presents the algorithm of generating five-axis scanning paths for curvilinear surfaces. A good measuring system should be appropriately accurate, i.e., properly matched to the expected accuracy of a manufactured product. In addition, when choosing a measuring device, its price should also be taken into account, because the costs of performing coordinate measurements affect the total costs of the entire production process of a product. In order to carry out measurements of a given product, apart from the choice of a measuring system for a measured object, it is also necessary to determine the appropriate measurement strategy for a given measurement task [5]. This strategy may take into account many factors that depend on an applied measuring system.

This article concerns selected issues related to measurements of free-form surfaces of products with the use of coordinate measuring machines. Free-form surfaces are represented by sets of control points which are used to determine the Bézier, B-spline and NURBS (Non-Uniform Rational B-Splines) surfaces [6]. The curvilinear surfaces are currently used, among others, in the automotive, aviation, shipbuilding and medical industries [1,7,8,9]. Moreover, free-form surfaces are also applied in architecture [10,11]. A free-form architectural design may be represented by circular arc snakes which are very useful because they enable the simplification of manufacturing of a complex free-form design [10]. The use of curvilinear surfaces is still increasing due to their aesthetic properties [12]. The first stage of measurements of free-form surfaces of products is the acquisition of measurement points [13], the number and the distribution of which depend on a used measurement method. Then, in the case of contact measurements, the probe radius correction process is carried out [14], the aim of which is to calculate points representing actual measurement points. In the next stages, the calculation of a substitute geometry [13], the best-fit of a measured surface to nominal one and the calculation of deviations of manufacturing of a given product [8] are conducted. On the basis of local deviations calculated at individual measurement points, it is possible to generate a map of deviations for the whole surface of a measured product with the use of, e.g., the finite element method. The obtained map of deviations is helpful during the compensation of errors of a manufacturing process of a considered product [13]. The number of measurement points and their distribution on a measured surface of an object significantly affect the accuracy of the best-fit process [1]. The coordinate measurements of curvilinear surfaces, due to their difficulty, are usually carried out by experienced operators of measuring systems [15]. In addition, measurements of free-form surfaces are very often carried out by using CMMs [16] due to their high accuracy and versatility.

The article presents the new method of determining the locations of scanning lines on curvilinear surfaces of products. Therefore, the paper concerns the first stage of coordinate measurements of free-form surfaces. The new method of generating measurement points on curvilinear surfaces was created mainly to improve the accuracy of measurements by increasing the chances of detecting the largest deviations of manufacturing. Additionally, the proposed method was developed to shorten the planning time of a measurement strategy.

The determination of locations of measurement points on measured surfaces is the fundamental element of strategies of contact coordinate measurements carried out with the use of coordinate measuring machines, which directly affects the accuracy of measurements and their time [17]. Therefore, the distances between measurement points and their number are the very important parameters in the coordinate metrology [12] and they are required when defining measurement strategies. The accurate measurements of complex surfaces require the use of methods of the distribution of measurement points reducing the uncertainty of measurements and fully reflecting the geometry of a measured surface of an object [18]. The applied method of the distribution of measurement points should enable the identification of the maximum deviation of a measured product [19]. Finding the optimal measurement strategy is the significant problem in the case of both surface reconstruction and its final measurement [20]. The strategy of selecting points has a significant impact on the accuracy of results of coordinate measurements [7]. In the case of measurements carried out with the use of CMMs, it is important to achieve a satisfactory level of measurement accuracy, taking into account the smallest possible number of measurement points [9], which especially in the case of measurements performed in the single point probing mode is directly related to time of a measurement task. Therefore, coordinate measurements should be performed by using inspection methods which require a small number of measurement points and enable obtaining sufficient information to calculate deviations of manufacturing a product [6]. The planning of a measurement strategy usually takes place after finishing a manufacturing process of a given product, during programming CMMs. This results in a significant extension of the preparation stage of a measurement program. Therefore, defining strategies of measurements performed with the use of coordinate measuring machines may also lead to a delay in a manufacturing process of a given product and thus to the generation of additional costs [21], reducing the productivity of a production process of a product. The process of strategy planning should be as automated as possible in order to speed up coordinate measurements. The next part of this article presents the state of the art regarding the methods of the distribution of measurement points on a measured surface of a product.

## 2. State of the Art in the Area of Determining the Location of Measurement Points

The strategies of selecting measurement points on a measured surface of an object are divided into three main categories [5]. The first of them is the so-called blind strategy, which is based only on the information about the nominal form of a measured area of a given product. In the case of the second group of methods, the positioning of measurement points is based on the knowledge of already selected points located on a surface of a measured object. The measurement strategies belonging to this category are called adaptive strategies. The adaptive distribution of measurement points in the coordinate metrology is possible, for example, on the basis of kriging models [22]. The last group includes measurement strategies that take into account the information about production processes of workpieces. However, the most common method is the uniform distribution of measurement points, which belongs to the first group. It provides reasonable accuracy but it requires a very large number of measurement points, which may make a measurement process inefficient. Moreover, uniformly distributed measurement points may inaccurately represent errors of a manufacturing process [17], causing that the maximum deviations of a manufactured workpiece may be omitted when conducting measurements. Many methods of determining locations of measurement points on measured surfaces of objects have been published so far. The next part of the second section of this article presents the selected achievements in the field of the distribution of measurement points, which are presented in other publications. The detailed analysis of these papers was the basis for further work, the results of which are presented in this paper, conducted to increase the efficiency of coordinate measurements.

Li et al. [7] presented the enhanced maximin distance method when selecting measurement points in order to simplify points cloud. The selection of points is conducted based on the angle computation between normal vectors calculated at measurement points. The proposed method is dedicated mainly to non-contact measurements.

An interesting model of determining the costs of a measurement process was proposed in the work [5]. The proposed model can be used to choose the optimal distance between measurement points.

Pagani and Scott [20] presented the strategy of the distribution of measurement points, which takes into account the length of a given curve and its complexity. For this purpose, the parameterization of a curve was calculated based on the length of an arc of a curve and B-wavelets basis functions, which reflected the complexity of a shape of a given curve. The paper presents the example, which indicates that more points are located in places of sudden changes in the shape of a curve, and the distance between points increases in the case of flat areas. The proposed method was also used in the case of surface measurements and compared to other methods of determining the positions of measurement points, e.g., a random method called Latin Hypercube Sampling (LHS). The LHS method is also used in the new method of the distribution of measurement points, what is described in the next sections of this article.

The work [9] presents the algorithm of the distribution of scanning lines on a free-form surface of a product. A substitute surface, representing a measured surface, is calculated on the basis of selected lines. In the case of inadequate accuracy of substitute geometry, additional scanning lines are included. The selection of the position of a scanning line results from the analysis of the complexity of a considered surface and the deviation calculated between a substitute surface and its nominal CAD (Computer-Aided Design) model. The presented algorithm does not take into account any other elements of a measurement strategy when determining the distribution of measurement points.

Raghunandan and Venkateswara Rao [23] showed that the arithmetic mean deviation (Ra) of an assessed profile is also a helpful parameter when determining the number of measurement points necessary for measuring the flatness deviation by using a coordinate measuring machine.

The method of the distribution of measurement points in the case of coordinate measurements of free-form surfaces performed with the use of a non-contact measuring system was proposed in the paper [15]. In the case of this method, the measurements are carried out along curves being cross-sections of a measured surface with the group of planes, and the distribution of control points is the result of, among others, curvature analysis.

Wang et al. [14] applied the uniform distribution of measurement points located along circular paths in order to conduct coordinate measurements of complex optical surfaces. The authors also pointed out that the element of a measurement strategy is an applied method of the probe radius correction process, presenting two methods for calculating corrected measurement points. The results of applying different methods of the probe radius compensation process may be used when planning the distribution of measurement points, as it was done in the case of the new method, what is explained in the next sections of this paper.

The authors of the article [16] proposed the use of genetic algorithms to optimize the motion path of a measuring probe cooperating with a coordinate measuring machine in order to increase the productivity of manufacturing processes.

The flow chart helpful during measurements of products with the use of coordinate measuring machines was presented in the work [19]. The authors pointed to different algorithms of the distribution of measurement points, which can be used in the case of measurements of form deviations. The authors recommend using, for example, the Hammersley method when measuring probes working in the single point probing mode are planned to be used.

The article [24] concerns a measurement strategy in the case of coordinate measurements of gears. The authors of this work proposed a minimum number of measurement points and their uniform distribution on the surface of a tooth of a gear. Unfortunately, in the case of free-form surfaces, the application of the uniform distribution of measurement points may not be a good idea especially when measurement time should not be too long.

Jiang et al. [25] presented the method of selecting measured cross-sections of a blade and measurement points located in each of measured cross-sections of an analyzed object. The work compared three selected methods of the distribution of measurement points. The most favorable results were achieved in the case of the chordal deviation method. Parallel planes were used to create measured cross-sections due to the type of a measured object.

The method of equi-parametric sampling presented in the work [12] was applied in order to measure a form deviation of curvilinear surfaces. The used method in not sensitive to significant changes of the shape of a measured surface of a product.

Rajamohan et al. [26,27] proposed new strategies of the distribution of measurement points based on the lengths of investigated curves and the areas of measured surfaces. Moreover, dominant points were used. The dominant points are the points at which the maximum local curvatures of both a curve and a free-form surface occur. In the case of a curvilinear surface, dominant points and corners of a surface form an initial set of points, which is used to create regions of a measured surface. In the next stage additional points are added to the created regions starting from the largest one.

The accuracy of an optical coordinate measuring system using the focus variation technique was analyzed in the paper [28]. The analysis was conducted with the use of four measurement strategies of the distribution of measurement points located on surfaces of measured test spheres and the performed analysis was based on the ISO 10360 standard.

The authors of the article [17] analyzed three selected methods of the distribution of measurement points located on three surfaces of products differing in shape and obtained by different manufacturing methods. The accuracy of, among others, the random distribution of measurement points for different number of points was analyzed. The so-called virtual sampling was applied during the analyses. The aim of the analyses was to select the method of the distribution of points, which includes the smallest possible number of points properly representing a measured surface. Measurement points were selected from the base group of points, which was obtained by means of coordinate measurements.

The estimation of the density function of the probability of geometrical deviations was based on the Parzen Windows technique in the paper [29]. The calculated function was used to identify fragments of a measured surface, which require additional measurements. The proposed method can be used in the case of measurements of both types of products characterized by regular geometric shapes and composed of free-form surfaces. The authors of the paper [30] proposed also the selection of measurement points for subsequent measurements in the case of coordinate measurements of the flatness deviation on the basis of the analysis of the function of the probability of deviations. The presented adaptive method was compared to the random distribution of measurement points and better results were obtained by using the adaptive method.

Wang et al. [31] compared selected methods of the distribution of points in the case of the surface metrology. The comparisons were made for three selected surfaces. The results of, among others, the uniform distribution of measurement points, the Halton method and adaptive methods were analyzed.

Two strategies of measurements are also shown in the work by ElKott and Veldhuis [32]. The paper concerns the selection of scanning lines on surfaces in the case of measurements conducted by a CMM. Two methods are proposed: The method considering maximum differences between a substitute model and a CAD model and the method analyzing the change of the average curvature of a free-form surface. Therefore, the presented methods are based on the analysis of the nominal geometry of a curvilinear surface.

In the work by ElKott et al. [33] four algorithms for sampling free-form surfaces are presented. They are based on: The uniform distribution of measurement points, the areas of surface patches, the list of surface patches sorted with regard to the calculated average curvature of a patch, the area of a surface patch and its average curvature. As in the case of the previous article, the presented methods use only the results of the geometry analysis of an investigated surface in order to distribute measurement points.

Barari [34] presents the algorithm of the distribution of points based on the information about the machining process. This information is obtained from simulations performed by using computer aided manufacturing software.

The authors of the referenced publications have not proposed an automated expert system supporting the user of a given coordinate measuring system in the stage of programming a measurement process and during defining a measurement strategy, which is the necessary element of all measurement programs. Moreover, other authors considered defining the distribution of measurement points independently from other elements of a strategy of contact measurements, what may contribute to increasing time of planning of a quality control process of curvilinear surfaces. Additionally, the mentioned publications do not present the algorithms of distributing measurement points that reduce measurement errors resulting from the probe radius correction procedure and occurring when conducting contact coordinate measurements.

Therefore, in this article, apart from the new method of determining the location of measurement points, the system of automated determination of suggested scanning lines and measurement points distributed along scanning lines, which is based on the fuzzy logic, was also proposed. The fuzzy logic has been already successfully used in the field of the coordinate measuring technique. The examples of its application are the processes of calculating corrected measurement points during the probe radius correction process [35], determining the orientation of a measuring probe [36], calculating the diameter of circular elements [37] and selecting the distribution of measurement points located on a free-form surface [38]. Unfortunately, the results presented in [38] have not been experimentally verified. The new method of determining the location of measurement points is much more advanced than the previous method presented by Magdziak and Ratnayake [38]. The proposed method includes more input parameters, deciding about the final distribution of measurement points, than the previous method. Moreover, one of the input parameters comes directly from a measurement process and it has a big impact on results of coordinate measurements of free-form surfaces. Additionally, the whole proposed method is much more universal than the previous method because it can be used by the users of coordinate measuring systems equipped with measuring probes working in different modes. Although the new method is intended primarily for measurements carried out in the single-point probing mode, one of its parts may also be used when programming a CMM working in the scanning mode. The results of applying the new method are scanning lines located on curvilinear surfaces and measurement points distributed along mentioned lines. The algorithm shown in [38] allows only to determine the positions of single measurement points in selected parts of a measured product, thus it enables obtaining limited information regarding the real form of a measured product.

The developed system can be used while preparing the technological process of a given product in the initial stages of its production process and therefore speeding up a measurement process of a given product. Moreover, the novelty of the proposed method of determining the location of measurement cross-sections is associated with the distribution of measurement points on curvilinear surfaces not only on the basis of the estimation of the geometrical complexity of a measured product but also taking into account the information about the accuracy of the probe radius correction process, which is one of the factors affecting the accuracy of coordinate measurements. The calculation of coordinates of corrected measurement points on the basis of coordinates of indicated measurement points, representing the center of a stylus tip during its contact with a surface of a measured object, is also the element of a strategy of contact coordinate measurements carried out with the use of, e.g., CMMs, which does not have to be considered independently from other components of a measurement strategy. Therefore, the presented method enables planning the distribution of measurement points together with selecting the probe radius correction method. This, in consequence, simplifies the determination of a measurement strategy, thereby affecting the increase in the efficiency of contact coordinate measurements and the productivity of the entire manufacturing process.

The following parts of this article include, among others, the algorithms of selecting scanning lines and measurement points distributed along selected measured cross-sections, results of simulation investigations for selected theoretical curvilinear surfaces of products and results of experimental investigations for one chosen free-form surface. The presented algorithms are intended primarily for CMMs equipped with measuring probes working in the single point probing mode, which are still often used in the industrial practice. Nevertheless, the new method of the distribution of measurement points can also be used by other measuring systems, e.g., CNC (Computer Numerical Control) machine tools equipped with contact measuring probes, which can be applied when measuring curvilinear surfaces of products. Numerical research was carried out with the use of selected computer-aided design software. Moreover, the MATLAB software was also applied.

## 3. New Method of the Distribution of Measurement Points on a Free-Form Surface

**Figure 1:**
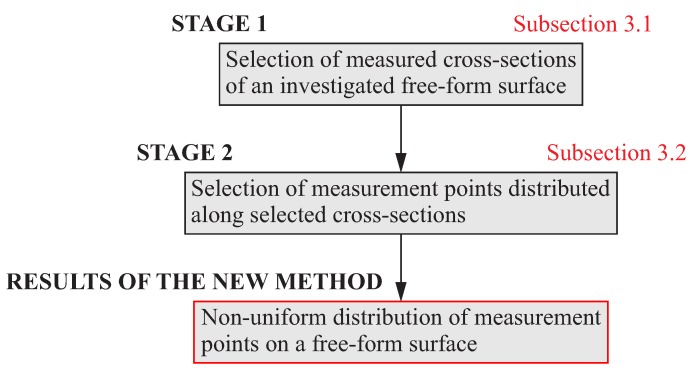
The general view of the proposed method of the distribution of measurement points.

The proposed method of the distribution of measurement points consists of two main parts. The assumption of the presented method is the use of measuring probes working in the single point probing mode, which are still used by coordinate measuring machines and CNC machine tools. The first part of the method is related to the choice of the positions of scanning lines, along which measurements are carried out by using a measuring probe of a CMM or a CNC machine tool. The second part of the method concerns the selection of the number of measurement points uniformly distributed along selected cross-sections of a curvilinear surface. The general view of the new method of the distribution of measurement points on a free-form surface is presented in Figure 1. The algorithms of the individual parts of the proposed method are presented in the following Section 3.1 and Section 3.2 of this paper.

The new method of defining the distribution of measurement points on free-form surfaces of objects is in accordance with the ISO 1101 standard. In the case of this standard, the following characteristics can be distinguished: The line profile and the surface profile. Both characteristics may refer to measurements of curvilinear surfaces. In the case of the line profile, the positions of measured cross-sections, along which measurements of a free-form surface are performed, should be determined. The need to define a specific orientation of a measured cross-section results from the position of the tolerance zone of the line profile read from a 2D drawing of a considered object composed of free-form surfaces. On the other hand, in the case of the surface profile, measurement points can be arbitrarily distributed on a free-form surface of an object to be measured. The proposed method concerns mainly measurements of the surface profile.

### 3.1. Algorithm of the Distribution of Scanning Lines

**Figure 2:**
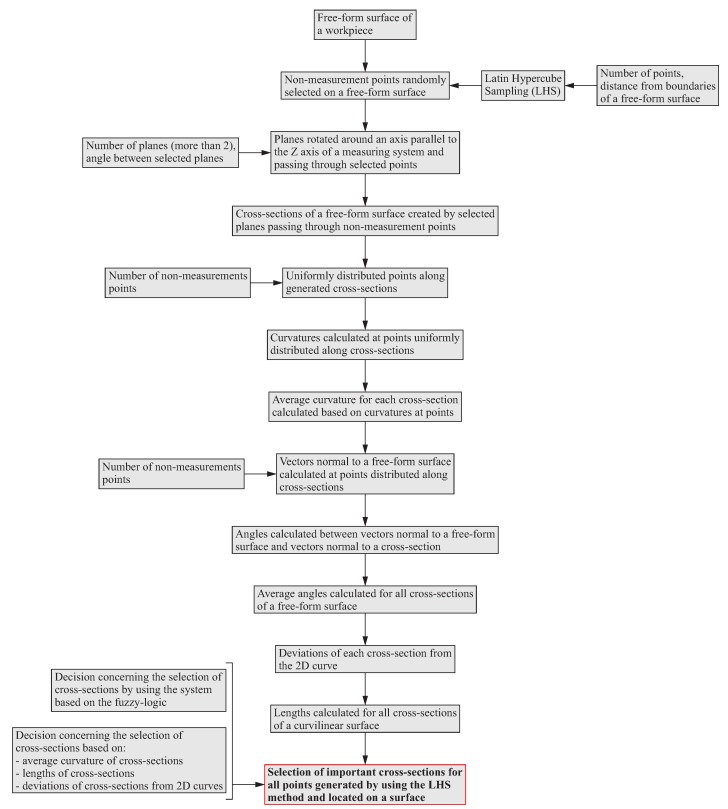
Algorithm of the distribution of scanning lines on a free-form surface of a workpiece.

This subsection presents the algorithm of the first part of the proposed method of the distribution of measurement points, which concerns the selection of measured cross-sections of free-form surfaces. The individual stages of the algorithm of the selection of a scanning line located on a curvilinear surface are presented in detail in Figure 2.

The first stage of the algorithm is the random distribution of points on a measured free-form surface of a product. This distribution is determined by using the Latin Hypercube Sampling (LHS) method. The LHS method is a statistical method which is used to generate random points on a surface in the case of the presented algorithm. The steps of using the LHS method are as follows:

- a free-form surface is divided into intervals in two directions;
- only one point in each row and column of a surface is selected.

Moreover, in the case of this method, the user of, e.g., a CMM must make decisions regarding the number of randomly distributed points and their positions in relation to boundaries of an analyzed curvilinear surface. The randomly generated points are not measurement points. In the next stage, a set of planes is created at the distributed points. The planes are used to form cross-sections of a measured surface. The number of planes and the angles between them are the input data processed by the considered algorithm and dependent on the decision of the CMM’s user. The third stage of the algorithm refers to the uniform distribution of points along the free-form cross-sections created in the second stage. The points generated in the third stage, like in the first stage of the algorithm, are not measurement points. Their purpose is only to determine places along analyzed cross-sections, in which further calculations will be carried out.

The next steps of the algorithm of the selection of scanning lines concern the calculation of:

- average curvatures for the generated cross-sections based on the values of curvatures at points uniformly distributed along the considered cross-sections;
- lengths of the created cross-sections of a measured free-form surface of a product;
- deviations of the analyzed cross-sections from the 2D curve.

The selection of a scanning line for points randomly distributed on a measured curvilinear surface is carried out by using a system based on the fuzzy logic. The input data for the calculations of membership functions of input parameters of the expert system is the information about the curvatures of the analyzed cross-sections, the lengths of the cross-sections of a free-form surface and the deviations of the cross-sections from 2D curves.

The dependence of the choice of the cross-section of a free-form surface, along which coordinate measurements should be made, on its length is the result of the need to distribute measurement points along the largest possible fragments of an analyzed curvilinear surface. This, in turn, increases the chances of locating measurement points in the areas of a measured surface of a product characterized by the largest values of form deviations.

The proposed method also provides measurement points in the parts of curvilinear surfaces characterized by the large curvature. Determining the locations of measurement points based on the curvature of a measured surface is the very often used approach in the coordinate metrology. The curvature is one of the parameters determining the distribution of measurement points applied in the methods proposed by the authors of the works [32,33]. Moreover, producers of measurement software also use such an approach. For instance, the Calypso software, which is very popular in the industry, also enables defining the distribution of points based on the analysis of the curvature of a surface of a measured product. In the proposed algorithm, an average curvature κ is calculated as the arithmetic mean of curvatures of a cross-section (Equation (1)). The curvatures of a cross-section $\kappa_{k}$ are measured at selected points uniformly distributed along an investigated section. The method of calculating an average curvature can be easily implemented in CAD software, which contributes to increasing the automation of computations and the reduction of the time of calculations.

(1) $$\kappa =\frac{\sum_{k=1}^{m}\kappa_{k}}{m}$$

where: *m*—a number of points, at which curvatures of a cross-section are measured.

The deviations of cross-sections of a free-form surface from the 2D curve are calculated based on the values of the arithmetic mean of the angles calculated between the vectors normal to a considered curvilinear surface and the vectors located in the planes which pass through sections of a measured surface. The mentioned vectors located in the planes are also normal to investigated cross-sections. The angles between the above mentioned vectors are calculated at selected points uniformly distributed along considered cross-sections of a curvilinear surface.

The number of points uniformly distributed along analyzed cross-sections of a surface, at which mentioned curvatures and angles (which are used for assessing the degree of deviation of a section of a surface from the 2D curve) are calculated, depends on the user of a coordinate measuring system. Therefore, the number of those points is the next input data for the algorithm of the selection of scanning lines, which results from the expected accuracy of numerical calculations.

The real coordinate measurements of curvilinear surfaces can be made by using, for instance, the 2D curve and the 3D curve measurement elements. The example of measurement software using such measurement elements for measuring curvilinear surfaces is the Calypso software package produced by the Carl Zeiss company. In the case of the 2D curve, vectors normal to a free-form curve at given points lie in one plane. In the algorithm of defining the positions of scanning lines, the deviation of a given cross-section from the 2D curve is taken into account due to the types of the methods of the probe radius compensation process that are to be used in the case of real coordinate measurements.

**Figure 3:**
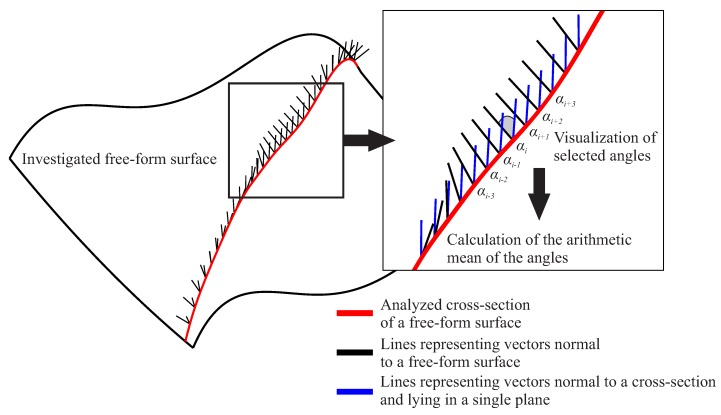
The calculation of the deviation of the selected cross-section from the 2D curve based on the arithmetic mean of the angles between normal vectors.

The calculation of the arithmetic mean of the angles ($(\sum_{k=1}^{n}\alpha_{k})/n$, where *n*—a number of points, at which angles $\alpha_{k}$ are calculated) is necessary because the assumed methods of the probe radius correction process, applied to find the final number of measurement points distributed along a selected measured cross-section of a free-form surface, give the best results for 2D profiles. The analysis of the accuracy of the probe radius correction process is included in the next algorithm of the proposed method regarding the distribution of measurement points along selected measured cross-sections. This algorithm is presented in the next subsection of the article (i.e., Section 3.2), which also includes the description of the applied methods of the probe radius compensation. Moreover, Figure 3 presents the process of the calculation of the deviation of one selected cross-section from the 2D curve.

The final stage of the algorithm concerns the selection of two measured cross-sections for each point randomly generated using the LHS method, located on an analyzed curvilinear surface. The measurements should be carried out along selected scanning lines.

In the case of the analysis of the line profile in accordance with the ISO 1101 standard, the scanning lines, selected from the set of all investigated cross-sections for performing real coordinate measurements, should be located in planes properly oriented with respect to a chosen base surface. The location of a scanning line should be the result of the analysis of a technical drawing of a measured object.

### 3.2. Algorithm of the Distribution of Measurement Points along Selected Scanning Lines

**Figure 4:**
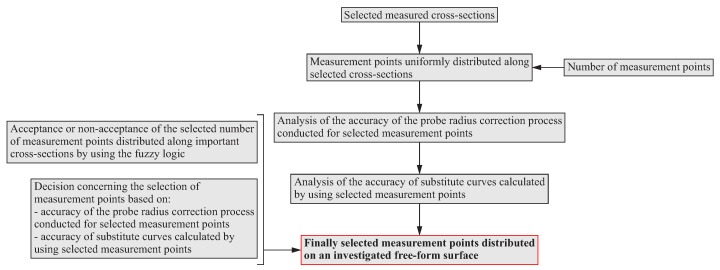
Algorithm of the distribution of measurement points along selected scanning lines.

After determining the distribution of scanning lines on a curvilinear surface of a measured object, the next stage of the proposed method concerns the decision about the number of measurement points, at which real coordinate measurements should be performed in the single point probing mode. The algorithm of the selection of the number of measurement points located along the selected scanning lines distributed on a curvilinear surface is presented in Figure 4.

In the initial stage of the algorithm, the uniform distribution of measurement points along the selected (by using the algorithm presented in Figure 2) scanning lines is generated. The initial number of measurement points depends on the user of a coordinate measuring machine and should be based on the user’s experience. The selected number of measurement points may result from the expected time required to perform a measurement process. The decision regarding the acceptance of the selected number of measurement points, analogously to the selection of scanning lines located on a free-form surface, is made again by using the expert system based on the fuzzy logic. Therefore, this system is intended to assist the user of a measuring system during the evaluation of the correctness of the selected number of measurement points. The effects of using the expert system are recommendations for not changing, reducing or increasing the pre-selected, based on the CMM operator’s experience, number of measurement points located on a measured free-form surface of an object.

The next steps of the algorithm of the distribution of measurement point along the selected scanning lines concern the analysis of the accuracy of:

- process of the probe radius correction carried out with the use of selected compensation methods;
- substitute curves fitted to the groups of measurement points generated along the scanning lines and representing a measured free-form surface.

The probe radius correction process is used in order to calculate the coordinates of corrected measurement points on the basis of indicated measurement points. Corrected points represent real contact points of a stylus tip with a surface of a measured product. Indicated points are the center points of a measuring stylus tip. There are many works by various authors, e.g., [39,40], dealing with the problem of the probe radius correction process. The system supporting the user in the selection of the number of measurement points examines the accuracy of two selected methods of the probe radius correction. The first one is based on the second degree Bézier curves, while the second one uses the fourth degree Bézier curves interpolating indicated measurement points to calculate coordinates of corrected points. The detailed equations enabling the calculations of corrected measurement points with the use of those two mentioned probe radius correction methods are presented in the work [41]. Moreover, the usefulness of the mentioned probe radius compensation methods in the case of measurements of curvilinear surfaces was analyzed by Kawalec and Magdziak in the paper [41].

The purpose of creating substitute curves for the selected scanning lines was, in turn, to check whether the declared number of points is adequate to accurately represent the geometric form of individual cross-sections of a free-form surface, along which a measurement process should be carried out.

The analysis of the accuracy of the probe radius correction process consisted of the analysis of the deviations between corrected measurement points, calculated using the above mentioned probe radius compensation methods, and scanning lines. In the case of substitute curves, the analysis of their accuracy was associated with the analysis of the deviations obtained by comparing substitute curves to a nominal model of a free-form surface. The accuracy of the probe radius compensation process and a substitute model increases as the mentioned deviations decrease.

The result of using the new method is the group of measurement points uniformly distributed along the selected scanning lines. This set of points creates the non-uniform distribution of measurement points for a whole measured curvilinear surface of a product. The presented method takes into account the various aspects of contact coordinate measurements. The method uses the information concerning not only the nominal form of a measured object but also regarding measurement errors, which may result from the application of the selected probe radius correction methods in the wrong places of an analyzed free-form surface.

## 4. Fuzzy-Logic-Based System

The expert system consisting of two parts and based on the fuzzy logic was developed. The fuzzy set theory has already been applied in order to calculate the number of points located on investigated surfaces [38]. Based on the results of the preliminary research [38], the use of this theory may improve the process of coordinate measurements of products. For example, the system based on the fuzzy logic may increase the efficiency of contact coordinate measurements by making the planning phase of measurements more automated. Other advantages of applying the fuzzy set theory are the short time of obtaining results of calculations regarding the distribution of measurement points on free-form surfaces and the possibility of using many various input parameters influencing the final distribution of points. Additionally, the expert system based on the fuzzy logic can be easily implemented in commercial measurement software and thus it can be used in industrial conditions.

The individual parts of the system, presented in detail in the Section 4.1 and Section 4.2, correspond to two algorithms of the presented method of defining the distribution of measurement points on free-form surfaces of objects. The first part of the system enables the selection of scanning lines on curvilinear surfaces of products from the set of generated cross-sections. In turn, the second one helps making the decision regarding the appropriate number of measurement points located along the selected, by using the previous part of the system, scanning lines. Both parts of the system were prepared by means of the Fuzzy Logic Toolbox of the MATLAB software. Those parts enable automated decision-making in relation to the selected elements of strategies of contact coordinate measurements. In the case of the Fuzzy Logic Toolbox, Mamdani-type fuzzy inference, not the Sugeno system, has been selected because it is very similar to the reasoning process of the users of, e.g., coordinate measuring systems and thus well-suited to a human input. Mamdani’s approach is very widespread in many industrial processes [42,43]. Moreover, the Mamdani systems are very intuitive [42].

### 4.1. Part of the System Used for the Selection of Scanning Lines

**Figure 5:**
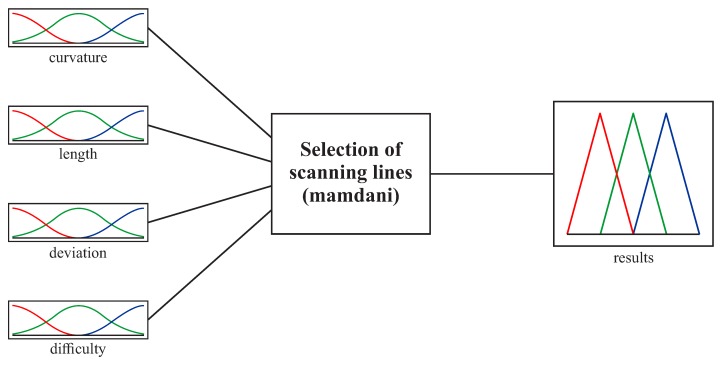
The structure of the first part of the expert system.

In this part of the system, the input parameters are the curvature of a considered cross-section of a free-form surface, its length and the deviation of a cross-section from the 2D curve. The proposed part of the expert system includes also the additional input parameter related to the evaluation of the difficulty of realizing a coordinate measurement along the selected cross-section of a considered curvilinear surface. The assessment of the complexity of a given measurement task should be carried out by the operator of a coordinate measuring machine. The aim of the application of the additional input parameter, compared to the proposed algorithm of the new method of the distribution of measurement points, for the first part of the expert system, responsible for selecting a scanning line, is to bring the proposed method closer to the real conditions of contact coordinate measurements. The difficulties of measurements along considered cross-sections may result from an applied probing system. The general structure of the first part of the developed expert system is illustrated in Figure 5.

**Figure 6:**
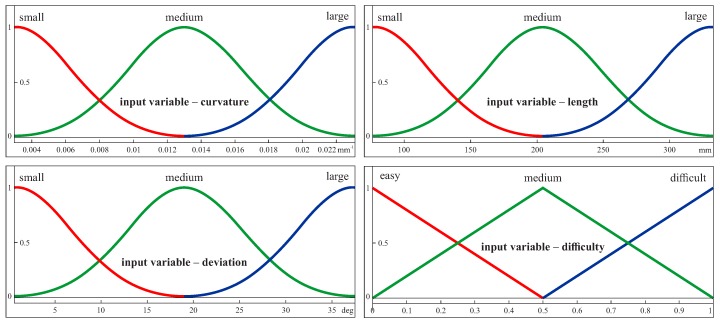
The membership functions for the input parameters in the case of the first free-form surface.

Three Gaussian membership functions (i.e., small, medium and large) were used for the first three input parameters. In the case of the parameter concerning the difficulty of a coordinate measurement, three triangular functions were used. Those functions for the individual input parameters are presented in Figure 6 in the case of the first considered curvilinear surface. The membership functions were created by using the Fuzzy Logic Toolbox of the MATLAB software (MathWorks, Natick, MA, USA). In the case of the Gaussian membership functions, one has to define the membership functions parameters, specified as the vector [σa], where σ is the standard deviation and *a* is the mean value [44]. For example, in the case of the first membership function (i.e., small) of the first input variable (i.e., curvature), the membership function parameters were equal to [0.00340.0030].

The ranges for those functions have been determined based on the differences between the extreme values of individual input parameters (i.e., a curvature, a length and a deviation from the 2D curve) calculated using the CATIA V5-6 software (Dassault Systèmes) during simulation research, the results of which are presented in the Section 6. The maximum and minimum values of curvatures, lengths and deviations were used to define the values of the x-axes of the plots of the membership functions. The MATLAB software does not require the normalized input data for the expert system. The user of this software can use the input values which are the direct results of the performed calculations.

**Figure 7:**
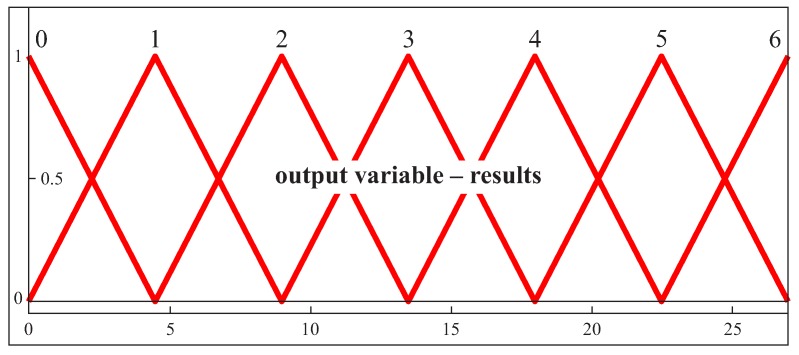
The membership functions for the output parameter in the case of the first free-form surface.

In the case of the output parameter, six triangular membership functions were used (Figure 7). The decision regarding the selection of scanning lines is made based on the value of the output parameter. Two cross-sections at each random point, generated using the LHS method, with the highest values of the output parameter are treated as scanning lines, along which coordinate measurements of an analyzed curvilinear surface should be made.

The system of the selection of scanning lines from generated cross-sections of a free-form surface is based on the rules, the selected examples of which are presented in Table 1.

### 4.2. Part of the System Used for the Selection of Number of Measurement Points

**Figure 8:**
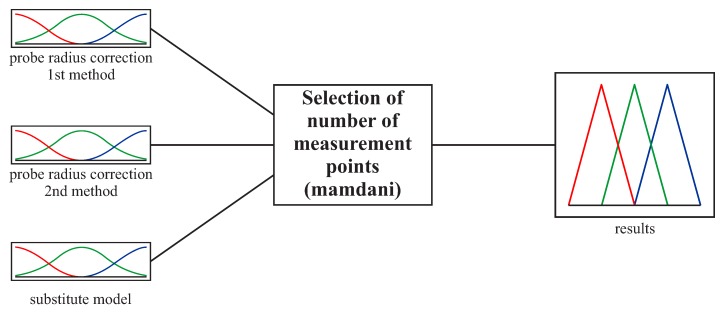
The structure of the second part of the expert system.

**Figure 9:**
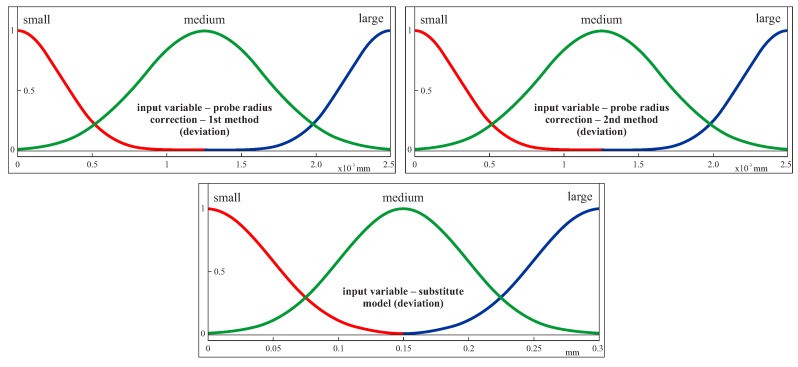
The membership functions for the input parameters in the case of both free-form surfaces and the second part of the expert system.

Three input parameters were used for the second part of the fuzzy logic based system. The first two are related to the accuracy of two probe radius correction methods based on the second and fourth degree Bézier curves. The accuracy of the probe radius compensation methods was determined on the basis of the average values of the deviations calculated between scanning lines and computed corrected measurement points. The third input parameter concerns the accuracy of substitute models of selected scanning lines. This accuracy was assessed based on the maximum deviations between offset curves and substitute curves interpolating selected groups of measurement points. The general view of the second part of the system is presented in Figure 8. Three Gaussian membership functions (small, medium and large) were used for all input parameters (Figure 9). The ranges for individual functions in the case of the second part of the fuzzy logic based system were determined based on the expected measurement accuracy.

**Figure 10:**
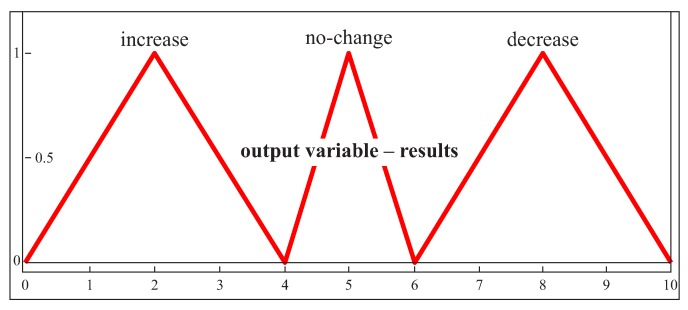
The membership functions for the output parameter in the case of both free-form surfaces and the second part of the expert system.

Three triangular membership functions were used for the output parameter (Figure 10). The value of the output parameter may belong to three different ranges. The decision regarding the proposed number of measurement points is made on the basis of this value. The number of measurement points may be reduced, increased or left unchanged.

The selected rules for the part of the system responsible for the assessment of the number of measurement points uniformly distributed along selected scanning lines are presented in Table 2.

## 5. Analyzed Workpieces Used during Simulation Investigations

**Figure 11:**
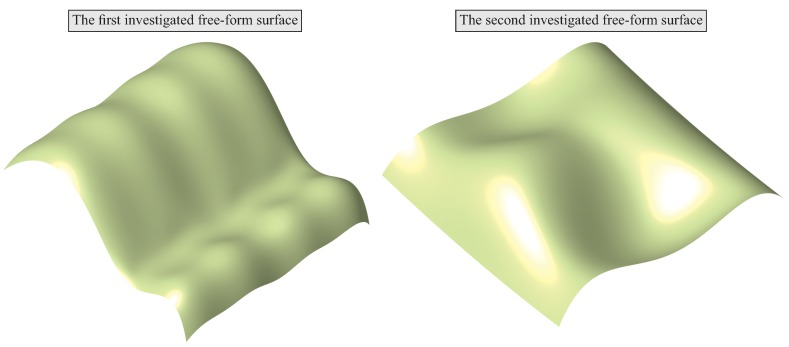
The investigated free-form surfaces of the workpieces.

The developed algorithms regarding the selection of scanning lines and the evaluation of a selected number of measurement points distributed along measured cross-sections were verified based on the examples of two theoretical products having upper curvilinear surfaces (Figure 11). The 3D models of the selected products were created by using the CATIA V5-6 CAD software due to its capabilities in the field of automation of modelling of 3D objects and automated calculations required for the created fuzzy logic based expert system.

## 6. Simulation Investigations

The simulation research concerned the selection of scanning lines and the number of measurement points distributed along selected scanning lines. The research was carried out for two selected free-form surfaces.

The macros enabling automatic generation of planes passing through points randomly distributed on the given surfaces in order to create cross-sections of the considered curvilinear surfaces and consequently scanning lines were prepared by means of the CATIA V5-6 software. Moreover, those macros enable the automated calculations of the average curvatures of considered cross-sections, their lengths and the deviations of cross-sections from 2D curves. Additionally, the developed macros enable the selection of maximum and minimum values among calculated values of curvatures, lengths of potential scanning lines and their deviations from 2D curves. The extreme values of curvatures, lengths and deviations of cross-sections from 2D curves in the case of the considered free-form surfaces were necessary to determine the membership function of the input parameters for the first part of the system based on the fuzzy logic, which assists a user when selecting scanning lines.

The simulation studies regarding the selection of scanning lines were carried out for 10 points randomly distributed on two free-form surfaces of the products. The points were distributed by using the Latin Hypercube Sampling method and the MATLAB software. The distance of points from the boundaries of the free-form surface equal to 20 mm was used. The applied distance should not be too small to avoid the positioning of scanning lines too close to the boundaries of the free-form surface. A scanning line located too close to the boundary may cause that the real contact coordinate measurements will be impossible to be carried out. The used distance depends on the experience of the user of a coordinate measuring system. For both considered free-form surfaces, the same group of the randomly distributed points was used. Four planes were generated at each random point and the angle between the neighboring planes was equal to 45°. The applied angle results from the necessity of using the uniform circular patterns of the analyzed cross-sections distributed in relation to the random points generated by means of the LHS method. The uniform patterns were used to simplify the algorithm of the distribution of scanning lines. The need for simplification results from the fact that the method is to be implemented in the industry. The planes were rotated around axes parallel to the Z axis of a coordinate measuring machine due to the assumption that a stylus of a measuring probe should be parallel to both the Z axis of a machine and the group of rotated planes. Such an orientation of a stylus will enable collision-free coordinate measurements of curvilinear surfaces whose shapes are similar to the shapes of the surfaces presented in Figure 11. Therefore, in the case of the proposed method of the distribution of measurement points, only planes parallel to the Z axis of a CMM were used to calculate cross-sections of the considered free-form surfaces. Thus, the proposed method is based on a different approach than that presented in the article [11]. Bartoň et al. [11] considered all planes in order to find movable planar profile curves, which may be used to generate parts of a given surface. The approach presented in the paper [11] cannot be used in the proposed method of the distribution of measurement points because of possible collisions which may occur during real coordinate measurements. The parallelism of planes and the stylus of a measuring probe results from the applied methods of the probe radius correction.

Moreover, 10 uniformly distributed points were applied to calculate the average curvatures for the generated cross-sections of the free-form surfaces. On the other hand, in the case of the calculations of the deviations of the curves from the 2D curve, the number of points at which the computations were made was equal to 30.

**Figure 12:**
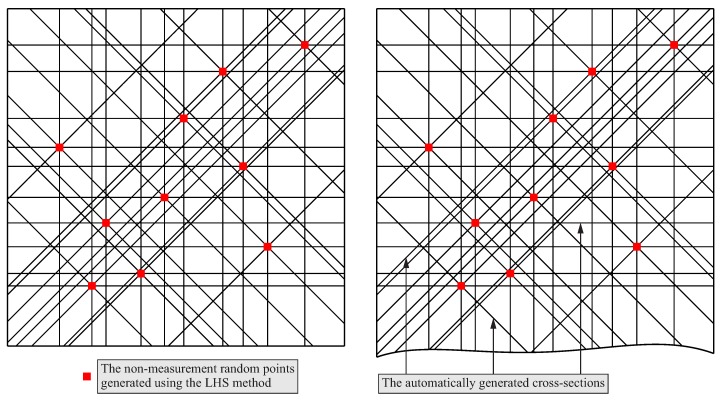
The generated cross-sections for two investigated free-form surfaces and the non-measurement points obtaind using the LHS method.

The automatically generated cross-sections for both considered curvilinear surfaces are presented in Figure 12. Those cross-sections were created using the macros prepared by means of the CATIA V5-6 software and the above mentioned values of the parameters. The scanning lines were selected from the visualized cross-sections by using the expert system based on the fuzzy logic. Moreover, Figure 12 presents the points randomly distributed on the analyzed free-form surfaces. The random points were generated by using the Latin Hypercube Sampling method and they were the basis for creating the cross-sections of the considered free-form surfaces.

Table 3 and Table 4 present the selected results concerning the values of the average curvatures of the generated cross-sections, the lengths of the cross-sections of the measured free-form surfaces of the products and the mean values of the angles being the basis for assessing the degree of the deviation of the analyzed cross-sections from the 2D curve. The medium level of difficulty of coordinate measurements was adopted. That data was the basis for choosing the scanning lines, along which coordinate measurements should be made, with the use of the first part of the expert system. Moreover, those tables include the information about the values of the output parameter calculated by using the first part of the system based on the fuzzy logic.

**Figure 13:**
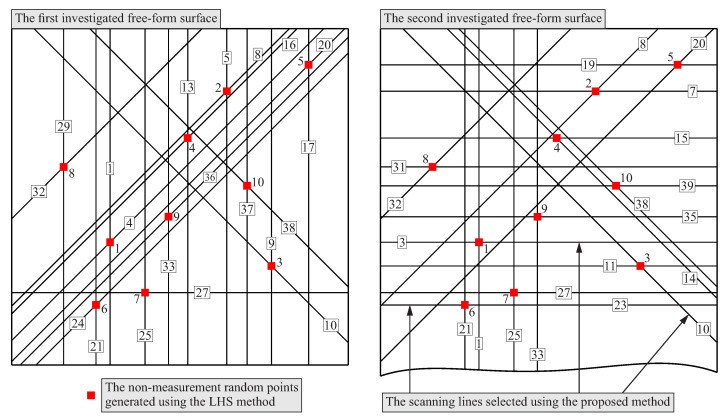
The selected scanning lines for two investigated free-form surfaces.

The completed calculations enabled the selection of scanning lines. The selected lines, along which coordinate measurements in the single point probing mode should be made, are presented in Figure 13 for both considered free-form surfaces.

The evaluation of the accuracy of the probe radius correction process performed by using two selected methods based on the Bézier curves and the accuracy of the substitute geometry representing individual cross-sections and scanning lines of a curvilinear surface was carried out by means of the computer-aided design software Rhinoceros. This software is dedicated mainly to modelling curves and free-form surfaces. In the first place the radius of the stylus tip had to be determined when working with the Rhinoceros software. This value is the fundamental result of the qualification process of probing systems in the case of contact coordinate measurements carried out with the use of coordinate measuring machines and it is necessary to conduct the probe radius correction process. The radius of a measuring tip was equal to 2.0 mm in the case of the simulation investigations. In the next step the offset curves of the selected scanning lines were calculated by using the commands of the Rhinoceros software. Those curves represented the indicated measurement points, for which their corrected equivalents had to be calculated.

The simulation research was carried out for 20 measurement points for both surfaces. The initially selected number of measurement points depends, similarly as in the case of the parameters necessary to generate the group of cross-sections of a surface, from which scanning lines are selected, on the decision of the user of an individual measuring system and the assessment of the time of coordinate measurements. The algorithm of the distribution of measurement points along selected scanning lines assumes choosing the starting number of measurement points, which may be done on the basis of the user’s experience. After selecting the initial number, the expert system may modify the applied number of measurement points and this is done independently of the user of a measuring system. The starting number of measurement points could be also chosen randomly but the experience of the user increases the probability of getting the right number of measurement points faster than with the random selection. This, in turn, may reduce the time of planning of a measurement strategy and speed up the start time of real coordinate measurements.

The probe radius correction process was carried out by using two selected methods based on the second and fourth degree curves for 20 indicated measurement points uniformly distributed along the offset curves. Subsequently, the calculated corrected measurement points were compared to the scanning lines. On the basis of this comparison, the values of the average deviations between the corrected points and the scanning lines were calculated for two methods of the probe radius compensation.

Moreover, the substitute models of the offset curves were evaluated for 20 measurement points uniformly distributed along the offset curves. The substitute curves were the B-Spline curves of the third degree interpolating selected measurement points. The cubic B-Spline curves are very often used during the process of modelling products. In the next stage, the substitute curves were compared to the offset curves of the individual scanning lines. The maximum values of deviations were calculated based on this comparison.

The maximum deviations and the mean deviations (determining the accuracy of the analyzed probe radius correction methods) were the basis for the assessment of the selection of the number of measurement points uniformly distributed along the scanning lines of both considered curvilinear surfaces. Table 5 and Table 6 present the average and maximum values of the deviations computed for the individual methods of the probe radius correction methods and the substitute curves. The calculations of those values were made for the selected scanning lines (i.e., for each point randomly located on a curvilinear surface, generated using the LHS method, the calculations were performed for two scanning lines selected by using the first part of the expert system presented in the Section 4.1). Moreover, those tables present the values of the output parameter and the recommendations concerning the applied number of measurement points.

## 7. Experimental Investigations

**Figure 14:**
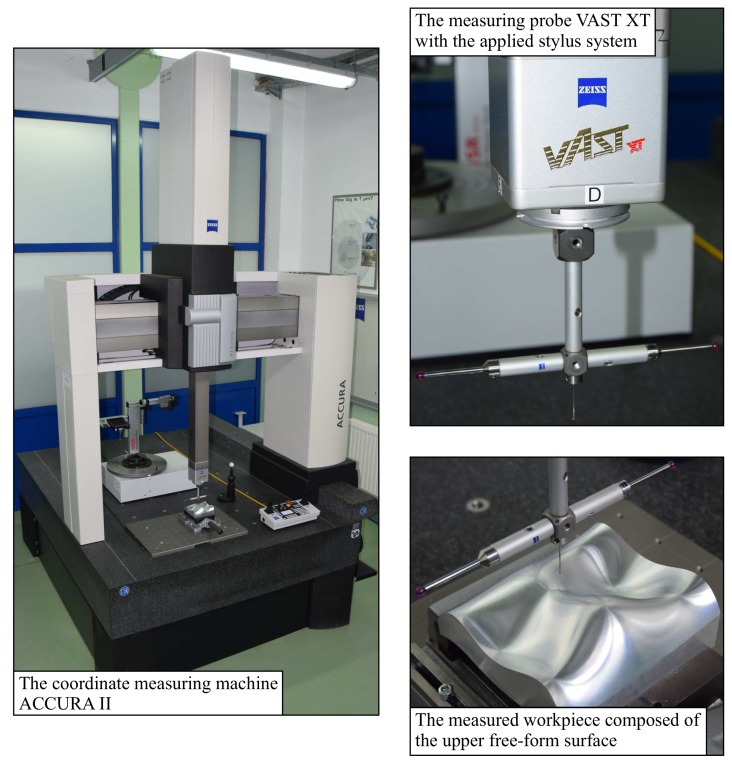
The applied measuring system used and the measured free-form surface.

The experimental research was carried out to verify the correctness of defining the positions of the scanning lines by using the proposed method of defining the location of measurement points on curvilinear surfaces of measured objects. The research was carried out for the second considered curvilinear surface (Figure 14). The measured object consisting of the upper analyzed free-form surface was made of an aluminum alloy by using the five-axis CNC machine tool DMU 100 monoBLOCK (DMG, Nagoya, Japan).

The aim of the experimental investigations was to check whether the scanning lines selected by using the new method of determining the distribution of measurement points enable detection of the deviations of the profile of the considered curvilinear surface that exceed the assumed tolerance. Therefore, the experimental investigations concerned the first part of the created method of defining the distribution of measurement points, which is related to the determination of the positions of scanning lines on a measured free-form surface. In order to check the correctness of the developed method, it was necessary to obtain the information regarding the form deviations of the considered curvilinear surface, which correspond as much as possible to the real deviations. The values of the form deviations were obtained by means of contact coordinate measurements made with the use of the ACCURA II coordinate measuring machine equipped with the VAST XT scanning measuring probe (Carl Zeiss, Jena, Germany). The measuring system applied when performing coordinate measurements of the analyzed free-form surface is presented in Figure 14. The accuracy parameters of the used system are as follows:

- $E_{L,MPE}=\left(1.6+L/333\right)\;\mu m$;
- $P_{FTU,MPE}=1.7\;\mu m$;
- $MPE_{Tij}=2.5\;\mu m$;
- $MPT_{\tau ij}=50.0\;s$;

where: $E_{L,MPE}$—a maximum permissible error of a length measurement; *L*—a measured length, mm; $P_{FTU,MPE}$—maximum permissible single-stylus form error; $MPE_{Tij}$—a maximum permissible scanning error; $MPT_{\tau ij}$—maximum permissible scanning test duration.

**Figure 15:**
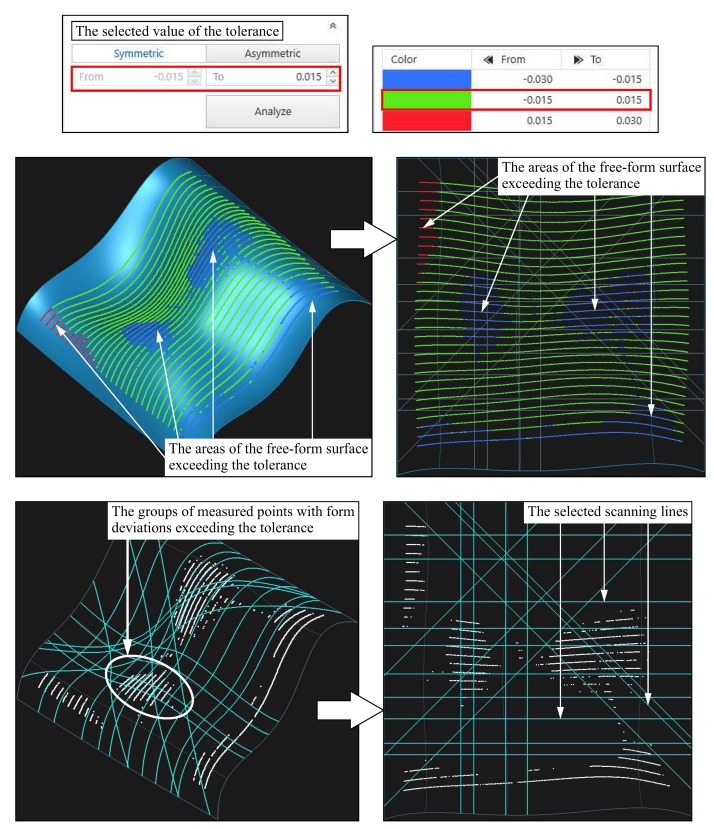
The distribution of form deviations on the measured free-form surface of the product together with the generated scanning lines and the selected value of the tolerance.

The number of scanning lines uniformly distributed along the measured surface during performing coordinate measurements was equal to 30. The scanning speed equal to 2.0 mm/s was used. The distance between measurement points was equal to 0.5 mm. The applied scanning parameters enabled measuring almost 7000 of measurement points. The application of the large number of measurement points was aimed at obtaining possibly the most accurate distribution of form deviations on the measured free-form surface of the product. The results were analyzed by using the Calypso and the Reverse Engineering software packages of the Carl Zeiss company. The results of the coordinate measurements are presented in Figure 15. The assumed value of the tolerance of the surface profile was equal to 0.03 mm and the tolerance zone was symmetrically located relative to the nominal curvilinear surface. Figure 15 presents also the scanning lines generated with the use of the first part of the proposed method, along which the coordinate measurements should be performed in the single point probing mode.

When analyzing the positions of the generated scanning lines, it can be observed that some of the measured cross-sections run through the fragments of the considered curvilinear surface, which are characterized by the form deviations exceeding the assumed value of the tolerance of the surface profile. Such positioning of measured cross-sections will increase the chances to detect the discrepancy between the actual form of the measured product and its design documentation during conducting real coordinate measurements.

In the case of some commercial methods of determining the location of measurement points on curvilinear surfaces, there is a large probability of not detecting deviations that exceed the accepted tolerance. The examples of such methods are:

- the method that enables the distribution of measurement points between two points located on a measured free-form surface and chosen by the operator of a CMM;
- the method of single measurement points, which are randomly selected on a considered curvilinear surface of a measured object by the user of a CMM.

**Figure 16:**
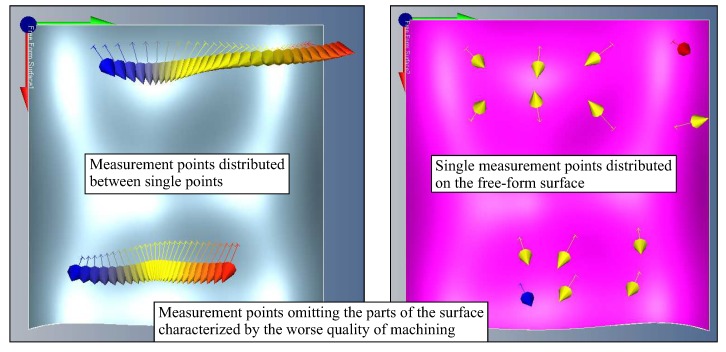
The selected methods of the distribution of measurement points available in the Calypso software.

Both above mentioned methods are available in the Calypso measurement software as parts of the Free-form surface measurement element. Figure 16 presents the usage of both methods. The vectors illustrated in Figure 16 are perpendicular to the free-form surface and they represent measurement points. The vectors were automatically generated by the Calypso measurement software. Measurement points were randomly chosen by the user of the applied measurement software. Based on the analysis of the distributions of measurement points, which were obtained using those methods, it can be noticed that there is the very big risk of omitting the parts of the measured free-form surface which are characterized by the worst quality of manufacturing when conducting measurements. This, in turn, may lead to the situation that the measured product will be treated as compatible with its technical specification when in fact its deviations exceed the assumed value of the tolerance.

## 8. Implementation of the System Based on the Fuzzy Logic in the Commercial Measurement Software

**Figure 17:**
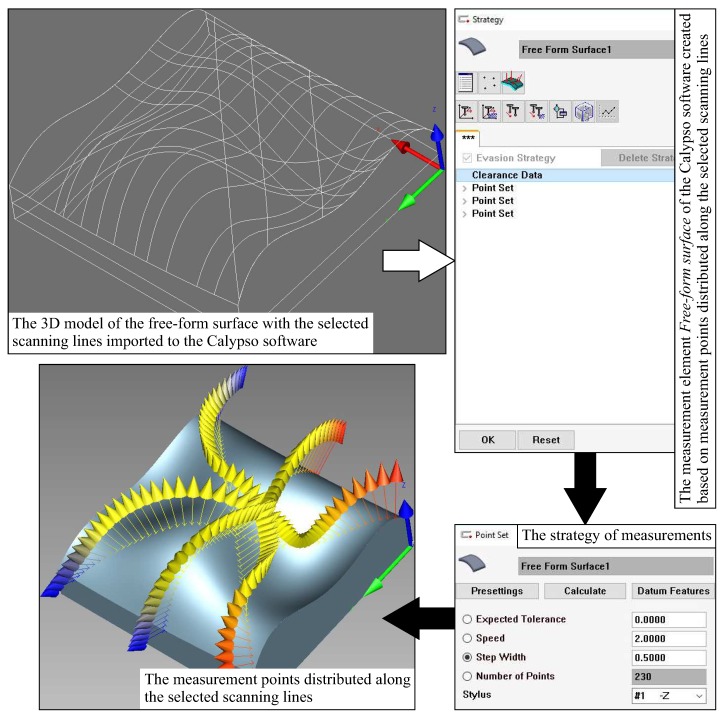
The most important stages of implementation of the created method in the commercial measurement software.

The created method of determining the positions of measurement points on a measured free-form surface can be implemented in commercial measurement software and thus used in the industry. In the case of the Calypso commercial measurement software, it is possible to define the distribution of measurement points on a curvilinear surface based on free-form curves selected by the user of the software. Those curves can be imported to measurement programs prepared by means of the above mentioned measurement software as the parts of the nominal CAD model of a measured object. The imported nominal model may include the measured cross-sections generated by using the first part of the developed method for determining the location of measurement points. Moreover, the user of a CMM can determine the number of measurement points uniformly distributed along the selected scanning lines by using the appropriate functions of the Calypso software. The adopted number of measurement points for individual scanning lines is the element of the strategy of coordinate measurements and may be the result of using the second part of the developed method for defining the distribution of measurement points. The stages of implementation of the created method in the selected commercial measurement software are presented in Figure 17.

## 9. Conclusions

The advantage of the developed method is the ability to determine the location of measurement points on curvilinear surfaces on the basis of several factors, thus taking into account different aspects of contact coordinate measurements. The presented method gives users of contact measuring systems the possibility of an individual approach when defining strategies of coordinate measurements. The employees of quality control departments can decide by themselves which aspects of measurements are more important. For example, in the case of some measurement tasks, the measurement time is the most significant parameter, and for other tasks the highest possible accuracy is the most important aspect when performing measurements. Moreover, the advantage of the presented method is not being the part of blind strategies.

The proposed fuzzy logic based expert system enables determining the distribution of measurement points on curvilinear surfaces of measured products not only on the basis of the decision of the operator of a contact measuring system. Therefore, the user of the presented method has less influence on the distribution of points on a measured free-form surface compared to the users of two presented commercial methods of determining the distribution of measurement points.

The obtained results of the experimental investigations confirm the usefulness of the proposed method of defining the distribution of measurement points. In addition, the undoubted advantages of the new method are the increase in the level of automation of defining measurement strategies and the possibility of the implementation of the developed method in commercial measurement software. Those advantages create, in turn, the chances to apply the developed method in various branches of the industry. Moreover, the high level of automation may increase the efficiency of coordinate measurements of curvilinear surfaces.

Further investigations may be aimed at the verification of the created method of the distribution of measurement points during measurements of other examples of free-form surfaces, not only theoretical ones but also occurring in products currently manufactured in the industry. In the case of more complex products, which are composed of many curvilinear surfaces, compared to these analyzed in this article, the proposed method could be used independently for each free-form surface. This would create the non-uniform distribution of measurement points for each surface and finally for a whole measured product. The new method does not take into account the generation of auxiliary movements of a measuring probe when moving a probe between individual measurement points, which may be the subject of further research. However, planning auxiliary movements can be relatively easily done manually by the operator of a CMM by means of functions of commercial measurement software because the proposed method does not require the use of advanced five-axis coordinate measuring machines as in the case of the algorithm presented in the paper [4]. Moreover, the users of most commercial measurement software can simulate a measurement program which is based on the distribution of measurement points generated by using the proposed method. The simulation enables the detection of potential collisions between a stylus tip of a probe and a measured object. Additionally, the further experimental investigations may also concern the second part of the developed method.

## Figures and Tables

**Table 1:** The selected rules for the first part of the developed system.

Curvature	Length	Deviation	Difficulty	Result
small	small	small	difficult	1
small	small	small	medium	2
small	small	small	easy	3
small	small	medium	difficult	0
small	small	medium	medium	1
small	small	medium	easy	2
small	small	large	difficult	0
small	small	large	medium	1
small	small	large	easy	2
small	medium	small	difficult	2
small	medium	small	medium	3
small	medium	small	easy	4
small	medium	medium	difficult	1
small	medium	medium	medium	2
small	medium	medium	easy	3
small	medium	large	difficult	0
small	medium	large	medium	1
small	medium	large	easy	2
small	large	small	difficult	3
small	large	small	medium	4
small	large	small	easy	5
small	large	medium	difficult	2
small	large	medium	medium	3

**Table 2:** The selected rules for the second part of the developed system.

Probe Radius Correction—1st Method	Probe Radius Correction—2nd Method	Substitute Model	Result
small	small	small	decrease
small	small	medium	decrease
small	small	large	no change
small	medium	small	decrease
small	medium	medium	decrease
small	medium	large	no change
small	large	small	no change
small	large	medium	no change
small	large	large	increase
medium	small	small	decrease
medium	small	medium	decrease
medium	small	large	no change
medium	medium	small	decrease
medium	medium	medium	no change
medium	medium	large	no change
medium	large	small	no change
medium	large	medium	no change
medium	large	large	increase
large	small	small	no change
large	small	medium	no change
large	small	large	increase
large	medium	small	no change
large	medium	medium	no change

**Table 3:** The selected results of simulation investigations being the basis for selecting the scanning lines for the first free-form surface.

Point	Cross-Section	Curvature, mm${}^{-1}$	Length, mm	Deviation, degree	Difficulty	Result
1	1	0.022	247.545	3.857	0.5	**21.8**
	2	0.008	208.244	22.423	0.5	11.2
	3	0.013	204.599	26.974	0.5	12.0
	4	0.015	298.126	17.423	0.5	17.8
2	5	0.023	247.657	5.553	0.5	21.6
	6	0.018	172.604	23.316	0.5	14.0
	7	0.020	208.571	32.389	0.5	13.4
	8	0.016	262.617	17.467	0.5	16.1
3	9	0.021	246.971	4.244	0.5	21.4
	10	0.016	299.152	18.525	0.5	17.9
	11	0.013	204.397	14.194	0.5	14.0
	12	0.010	176.200	16.536	0.5	12.5
4	13	0.020	246.746	4.899	0.5	20.8
	14	0.015	252.293	18.580	0.5	15.1
	15	0.003	201.231	6.238	0.5	12.8
	16	0.016	269.904	16.918	0.5	16.6

**Table 4:** The selected results of simulation investigations being the basis for selecting the scanning lines for the second free-form surface.

Point	Cross-Section	Curvature, mm${}^{-1}$	Length, mm	Deviation, degree	Difficulty	Result
1	1	0.014	222.527	14.010	0.5	15.2
	2	0.012	206.815	16.586	0.5	13.8
	3	0.014	247.648	11.675	0.5	17.7
	4	0.007	279.070	23.548	0.5	12.4
2	5	0.017	229.304	25.655	0.5	15.1
	6	0.018	188.269	25.37	0.5	14.6
	7	0.019	232.346	3.421	0.5	22.2
	8	0.010	263.425	20.638	0.5	15.4
3	9	0.013	212.116	12.813	0.5	15.1
	10	0.011	298.818	16.755	0.5	17.5
	11	0.015	241.833	20.631	0.5	16.1
	12	0.014	184.554	20.349	0.5	13.8
4	13	0.017	233.728	19.435	0.5	18.0
	14	0.013	278.197	9.091	0.5	20.5
	15	0.019	239.164	19.862	0.5	19.1
	16	0.010	267.040	21.276	0.5	15.0

**Table 5:** The results of the second part of the simulation investigations and the recommendations regarding the number of measurement points located along the selected measured cross-sections for the first free-form surface.

Point	Cross-Section	Deviation—1st Method of the Probe Radius Correction, mm	Deviation—2nd Method of the Probe Radius Correction, mm	Deviation—Substitute Model, mm	Result	Recommendation
1	1	0.002011	0.000705	0.260878	3.7	increase
	4	0.001353	0.000668	0.296815	5.2	no change
2	5	0.002174	0.000879	0.287335	2.4	increase
	8	0.000634	0.000131	0.055623	7.9	decrease
3	9	0.001378	0.000242	0.125011	7.8	decrease
	10	0.001360	0.000818	0.328597	5.1	no change
4	13	0.001396	0.000357	0.151204	7.6	decrease
	16	0.000647	0.000134	0.045928	7.9	decrease
5	17	0.001347	0.000220	0.125403	7.9	decrease
	20	0.001730	0.000944	0.437777	4.6	no change
6	21	0.001605	0.000394	0.177740	7.4	decrease
	24	0.001103	0.000316	0.145900	7.7	decrease
7	25	0.001471	0.000293	0.124325	7.8	decrease
	27	0.000524	0.000095	0.077839	7.9	decrease
8	29	0.002449	0.001109	0.332622	2.1	increase
	32	0.000228	0.000095	0.027453	7.9	decrease
9	33	0.001360	0.000358	0.149707	7.7	decrease
	36	0.000947	0.000182	0.144307	7.9	decrease
10	37	0.002240	0.000923	0.306250	2.3	increase
	38	0.000517	0.000144	0.081611	7.8	decrease

**Table 6:** The results of the second part of the simulation investigations and the recommendations regarding the number of measurement points located along the selected measured cross-sections for the second free-form surface.

Point	Cross-Section	Deviation—1st Method of the Probe Radius Correction, mm	Deviation—2nd Method of the Probe Radius Correction, mm	Deviation—Substitute Model, mm	Result	Recommendation
1	1	0.000191	0.000143	0.073943	7.85	decrease
	3	0.000342	0.000122	0.057719	7.9	decrease
2	7	0.000365	0.000049	0.051129	7.94	decrease
	8	0.000149	0.000095	0.042674	7.93	decrease
3	10	0.000304	0.000097	0.113102	7.93	decrease
	11	0.000296	0.000116	0.040633	7.92	decrease
4	14	0.000164	0.000058	0.055943	7.94	decrease
	15	0.000410	0.000101	0.050415	7.9	decrease
5	19	0.000351	0.000090	0.0485772	7.92	decrease
	20	0.000147	0.000102	0.070310	7.89	decrease
6	21	0.000105	0.000075	0.069746	7.91	decrease
	23	0.000216	0.000062	0.036623	7.95	decrease
7	25	0.000213	0.000113	0.057528	7.91	decrease
	27	0.000286	0.000126	0.036728	7.92	decrease
8	31	0.000390	0.000069	0.051482	7.93	decrease
	32	0.000095	0.000046	0.012794	7.96	decrease
9	33	0.000252	0.000049	0.048382	7.95	decrease
	35	0.000387	0.000127	0.062302	7.89	decrease
10	38	0.000173	0.000062	0.046361	7.94	decrease
	39	0.000352	0.000123	0.051950	7.91	decrease

## References

[1] Mehrad V., Xue D., Gu P. (2014). Robust localization to align measured points on the manufactured surface with design surface for freeform surface inspection. Comput. Aided Des..

[2] Zapico P., Patiño H., Valiño G., Fernández P., Rico J.C. (2019). CNC centralized control for digitizing freeform surfaces by means of a conoscopic holography sensor integrated in a machining centre. Precis. Eng..

[3] Li Y., Nomula P.R. (2015). Surface-opening feature measurement using coordinate-measuring machines. Int. J. Adv. Manuf. Technol..

[4] Hu P., Zhou H., Chen J., Lee C., Tang K., Yang J., Shen S. (2018). Automatic generation of efficient and interference-free five-axis scanning path for free-form surface inspection. Comput. Aided Des..

[5] Moroni G., Petrò S. (2014). Optimal inspection strategy planning for geometric tolerance verification. Precis. Eng..

[6] Li Y., Gu P. (2004). Free-form surface inspection techniques state of the art review. Comput. Aided Des..

[7] Li T., Gao L., Pan Q., Li P. (2018). Free-form surface parts quality inspection optimization with a novel sampling method. Appl. Soft Comput..

[8] Ren M., Cheung C., Kong L., Wang S. (2015). Quantitative Analysis of the Measurement Uncertainty in Form Characterization of Freeform Surfaces based on Monte Carlo Simulation. Procedia CIRP.

[9] Ren M., Kong L., Sun L., Cheung C. (2017). A Curve Network Sampling Strategy for Measurement of Freeform Surfaces on Coordinate Measuring Machines. IEEE Trans. Instrum. Meas..

[10] Bartoň M., Shi L., Kilian M., Wallner J., Pottmann H. (2013). Circular Arc Snakes and Kinematic Surface Generation. Comput. Graph. Forum.

[11] Bartoň M., Pottmann H., Wallner J. (2014). Detection and reconstruction of freeform sweeps. Comput. Graph. Forum.

[12] Tesfay G., Rajendra R. (2017). Global Form Deviation Evaluation of Free-Form Surface using Coordinate Measuring Machine. IJMPERD.

[13] Lalehpour A., Barari A. (2017). Developing skin model in coordinate metrology using a finite element method. Measurement.

[14] Wang X., Xian J., Yang Y., Zhang Y., Fu X., Kang M. (2017). Use of coordinate measuring machine to measure circular aperture complex optical surface. Measurement.

[15] Liu H., Wang Y., Huang X., Xue L. (2013). Isoplanar-based adaptive sampling for model-unknown sculptured surface coordinate metrology using non-contact probe. Int. J. Adv. Manuf. Technol..

[16] Qu L., Xu G., Wang G. (1998). Optimization of the measuring path on a coordinate measuring machine using genetic algorithms. Measurement.

[17] Lalehpour A., Berry C., Barari A. (2017). Adaptive data reduction with neighbourhood search approach in coordinate measurement of planar surfaces. J. Manuf. Syst..

[18] Sun L., Ren M., Yin Y. (2017). Domain-specific Gaussian process-based intelligent sampling for inspection planning of complex surfaces. Int. J. Prod. Res..

[19] Álvarez B.J., Cuesta E., Martínez S., Barreiro J., Fernández P., Hinduja S., Li L. (2010). Implementation of decision rules for CMM sampling in a KBE system. Proceedings of the 36th International MATADOR Conference.

[20] Pagani L., Scott P.J. (2016). A sampling strategy based on B-wavelets decomposition. Procedia CIRP.

[21] Anagnostakis D., Ritchie J., Lim T., Sung R., Dewar R. (2018). Automated coordinate measuring machine inspection planning knowledge capture and formalization. J. Comput. Inf. Sci. Eng..

[22] Ascione R., Moroni G., Petrò S., Romano D. (2013). Adaptive inspection in coordinate metrology based on kriging models. Precis. Eng..

[23] Raghunandan R., Venkateswara Rao P. (2008). Selection of sampling points for accurate evaluation of flatness error using coordinate measuring machine. J. Mater. Process. Technol..

[24] Gao C.H., Cheng K., Webb D. (2004). Investigation on sampling size optimisation in gear tooth surface measurement using a CMM. Int. J. Adv. Manuf. Technol..

[25] Jiang R.-S., Wang W.-H., Zhang D.-H., Wang Z.-Q. (2016). A practical sampling method for profile measurement of complex blades. Measurement.

[26] Rajamohan G., Shunmugam M.S., Samuel G.L. (2011). Effect of probe size and measurement strategies on assessment of freeform profile deviations using coordinate measuring machine. Measurement.

[27] Rajamohan G., Shunmugam M.S., Samuel G.L. (2011). Practical measurement strategies for verification of freeform surfaces using coordinate measuring machines. Metrol. Meas. Syst..

[28] Sun W., Claverley J.D. (2015). Verification of an optical micro-CMM using the focus variation technique: Aspects of probing errors. CIRP Ann.-Manuf. Technol..

[29] Barari A., ElMaraghy H.A., Knopf G.K. (2007). Search-guided sampling to reduce uncertainty of minimum deviation zone estimation. J. Comput. Inf. Sci. Eng..

[30] Martins T.C., Tsuzuki M.S.G., Takimoto R.Y., Barari A., Gallo G.B., Garcia M.A.A., Tiba H. (2014). Algorithmic iterative sampling in coordinate metrology plan for coordinate metrology using dynamic uncertainty analysis. Proceedings of the 2014 12th IEEE International Conference on Industrial Informatics (INDIN).

[31] Wang J., Jiang X., Blunt L.A., Leach R.K., Scott P.J. (2012). Intelligent sampling for the measurement of structured surfaces. Meas. Sci. Technol..

[32] ElKott D.F., Veldhuis S.C. (2005). Isoparametric line sampling for the inspection planning of sculptured surfaces. Comput. Aided Des..

[33] ElKott D.F., Elmaraghy H.A., Elmaraghy W.H. (2002). Automatic sampling for CMM inspection planning of free-form surfaces. Int. J. Prod. Res..

[34] Barari A. (2013). Inspection of the machined surfaces using manufacturing data. J. Manuf. Syst..

[35] Wozniak A., Mayer R., Balazinski M., Reformat M., Berthold M.R. (2007). Application of fuzzy knowledge base for corrected measured point determination in coordinate metrology. Proceedings of the NAFIPS 2007—2007 Annual Meeting of the North American Fuzzy Information Processing Society.

[36] Beg J., Shunmugam M.S. (2003). Application of fuzzy logic in the selection of part orientation and probe orientation sequencing for prismatic parts. Int. J. Prod. Res..

[37] Cappetti N., Naddeo A., Villecco F. (2016). Fuzzy approach to measures correction on coordinate measuring machines: The case of hole-diameter verification. Measurement.

[38] Magdziak M., Ratnayake R.M.C. Contact Coordinate Measurements of Free-form Surfaces: A FIS for Optimal Distribution of Measurement Points. Proceedings of the 2018 IEEE International Conference on Industrial Engineering and Engineering Management.

[39] Ahn H.K., Kang H., Ghim Y.-S., Yang H.-S. (2019). Touch probe tip compensation using a novel transformation algorithm for coordinate measurements of curved surfaces. Int. J. Precis. Eng. Manuf..

[40] Chen S., Wu C., Xue S., Li Z. (2018). Fast registration of 3D point clouds with offset surfaces in precision grinding of free-form surfaces. Int. J. Adv. Manuf. Technol..

[41] Kawalec A., Magdziak M. (2017). The selection of radius correction method in the case of coordinate measurements applicable for turbine blades. Precis. Eng..

[42] MathWorks Comparison of Sugeno and Mamdani Systems. uk.mathworks.com/help/fuzzy/comparison-of-sugeno-and-mamdani-systems.html.

[43] Ratnayake R.M.C. (2015). Estimation of Maximum In-Service Inspection Intervals Based on Risk: A Fuzzy Logic Based Approach. Int. J. Perform. Eng..

[44] MathWorks Documentation of the MATLAB Software Regarding the Gaussian Membership Function. uk.mathworks.com/help/fuzzy/gaussmf.html.

